# *In Silico* Head-to-Head Comparison of Insulin Glargine 300 U/mL and Insulin Degludec 100 U/mL in Type 1 Diabetes

**DOI:** 10.1089/dia.2020.0027

**Published:** 2020-07-27

**Authors:** Michele Schiavon, Roberto Visentin, Clemens Giegerich, Jochen Sieber, Chiara Dalla Man, Claudio Cobelli, Thomas Klabunde

**Affiliations:** ^1^ Department of Information Engineering, University of Padova, Padova, Italy.; ^2^ Translational Disease Modeling, R&D Digital and Data Sciences, Sanofi-Aventis Deutschland GmbH, Frankfurt am Main, Germany.; ^3^ Medical Affairs Diabetes Care EMEA, Becton, Dickinson and Company.

**Keywords:** Basal insulin, Mathematical modeling, Pharmacokinetics, Pharmacodynamics, Virtual trial

## Abstract

***Background:*** Second-generation long-acting insulin glargine 300 U/mL (Gla-300) and degludec 100 U/mL (Deg-100) provide novel basal insulin therapies for the treatment of type 1 diabetes (T1D). Both offer a flatter pharmacokinetic (PK) profile than the previous generation of long-acting insulins, thus improving glycemic control while reducing hypoglycemic events. This work describes an *in silico* head-to-head comparison of the two basal insulins on 24-h glucose profiles and was used to guide the design of a clinical trial.

***Materials and Methods:*** The Universities of Virginia (UVA)/Padova T1D simulator describes the intra-/interday variability of glucose-insulin dynamics and thus provides a robust bench-test for assessing glucose control for basal insulin therapies. A PK model describing subcutaneous absorption of Deg-100, in addition to the one already available for Gla-300, has been developed based on T1D clinical data and incorporated into the simulator. One hundred *in silico* T1D subjects received a basal insulin dose (Gla-300 or Deg-100) for 12 weeks (8 weeks uptitration, 4 weeks stable dosing) by morning or evening administration in a basal/bolus regimen. The virtual patients were uptitrated to their individual doses with two different titration rules.

***Results:*** The last 2-week simulated continuous glucose monitoring data were used to calculate various outcome metrics for both basal insulin treatments, with primary outcome being the percent time in glucose target (70–140 mg/dL). The simulations show no statistically significant difference for Gla-300 versus Deg-100 in the main endpoints.

***Conclusions:*** This work suggests comparable glucose control using either Gla-300 or Deg-100 and was used to guide the design of a clinical trial intended to compare second-generation long-acting insulin analogues.

## Introduction

Subcutaneous (sc) basal and prandial insulin administration is a key component of multiple daily injection (MDI) therapy in patients with type 1 diabetes (T1D) and with insulin-dependent type 2 diabetes (T2D). In the last decades, advances have been made in developing new basal and prandial insulin analogues aiming to mimic endogenous insulin secretion of healthy subjects. In particular, second-generation long-acting (basal) insulin analogues exhibit flatter pharmacokinetic (PK) and pharmacodynamic (PD) profiles, with an insulin dosing frequency not inferior than once daily, thus targeting an action duration exceeding 24 h.^1,2^

Insulin glargine 300 U/mL (Gla-300) and degludec 100 U/mL (Deg-100) represent the second generation of long-acting insulin analogues, with respect to insulin glargine 100 U/mL (Gla-100) and insulin detemir 100 U/mL (Det-100), respectively, showing a more prolonged and stable insulin profile over 24 h.

This is achieved through different mechanisms of protraction.^3,4^ Insulin Gla-300, similarly to Gla-100, rapidly precipitates at the injection site, due to a low solubility at physiological pH, then, it is absorbed into plasma with a slower rate than Gla-100 thanks to its higher concentrated formulation.^5^ Insulin Deg-100, thanks to the acylation of a long-chain fatty acid to the insulin molecule, forms a soluble multihexamer depot at the injection site, with a higher molecular weight than Det-100, and then reversibly binds to albumin. As a consequence of the high affinity to albumin, it is immediately and almost completely (98%–99%) bound to circulating albumin, so that the free active insulin concentration, which is the one available to the insulin receptor, is kept very low.^6,7^

In euglycemic clamp studies, with respect to insulin Gla-100, both Gla-300 and Deg-100 showed prolonged and more stable PK and PD profiles.^8–10^ In addition, for both the second-generation long-acting insulins, less hypoglycemic events and noninferiority in terms of HbA1c reduction were shown.^11,12^ However, only a few studies compared Gla-300 versus Deg-100 providing controversial results: a lower day-to-day and within-day variability in PD (evaluated from a glucose infusion rate in a euglycemic clamp study) of Deg-100 compared to Gla-300 was reported in Heise et al.,^13^ while a lower within-day variability in PD and a more evenly distributed PK profile was shown for Gla-300 compared to Deg-100 in Bailey et al.^14^ However, the features of the two protocols may explain, at least in part, these somewhat different results.^15^

More similarities than differences have also been observed in insulin-naive T2D patients comparing both the second-generation long-acting insulins in a randomized head-to-head clinical trial with respect to efficacy on HbA1c reduction and hypoglycemic events.^16^ So far, no head-to-head clinical trial has been conducted in patients with T1D in daily life conditions comparing the effect of Deg-100 versus Gla-300 on glycemic control and glucose variability using continuous glucose monitoring (CGM). Furthermore, a clinical head-to-head evaluation of the benefits and risks of different titration rules and/or insulin dosing schedules is currently still missing. Such a study would be very useful to assess Deg-100 versus Gla-300 performance in daily life conditions and/or to optimize their dosing. However, this would require an extensive, and consequently, a costly and time-consuming, clinical trial to make any possible differences visible for different insulins/dosing schedules/titration rules.

Computer simulations can be extremely helpful to test *in silico* different treatments under several experimental conditions in a time- and cost-effective way and are increasingly used by pharmaceutical companies to support decision-making. We recently equipped the Universities of Virginia (UVA) and Padova T1D simulator (T1DS)^17^ with a model of sc absorption of long-acting insulin glargine^18^ (Gla-100 and Gla-300) allowing to perform *in silico* testing of MDI therapies.^19^ In this work, we first developed a PK model of insulin Deg-100, based on individual patient-level clinical data, and incorporated it into the UVA/Padova T1DS^19^; then, we used the UVA/Padova simulator to perform an *in silico* (virtual) trial evaluating the benefits/risks of insulin Gla-300 and Deg-100 administration in patient with T1D to support the design of a head-to-head clinical trial comparing the two insulin analogues (NCT04075513).

## Research Design and Methods

### The UVA/Padova T1DS

The UVA/Padova T1DS^17,20^ is a tool accepted by the U.S. Food and Drug Administration as a substitute for preclinical trials of certain insulin treatments, such as artificial pancreas,^21^ insulin analogues,^22^ and glucose sensors.^23^ It consists of a model of glucose–insulin–glucagon dynamics and a population of *in silico* T1D subjects (100 adults, 100 adolescents, and 100 children). The latest version of the simulator^17^ has been updated with a series of novelties, among which a recently developed model of diurnal glucose variability,^24^ which extends its domain of validity from single- to multiple-meal scenarios,^25^ and the sc insulin delivery,^26^ now allowing also to describe commercially available fast-acting insulin analogues.

In addition, a model of sc absorption of long-acting insulin glargine (Gla-100 and Gla-300) has been recently developed^18^ and incorporated into the T1DS,^19^ allowing to simulate MDI therapies. In the following sections, a model of insulin degludec PK is presented and incorporated into the simulator thus extending the simulator MDI module to this recently developed long-acting insulin analogue. To do so, a database of T1D subjects receiving multiple doses of Deg-100 is used.^14^

### Development of insulin Deg-100 PK model

#### Database

The data used for model development come from a randomized, double-blind, two-treatment, two-period, two-sequence crossover trial comparing multiple-dose administration of insulin glargine (Gla-300) versus degludec (Deg-100) (EudraCT no. 2015-004843-38).^14^ Briefly, 48 T1D subjects, grouped in two cohorts (cohort 1 [*n* = 24]: 22 males, age = 44 ± 10 years; BMI = 25.4 ± 2.5 kg/m^2^; cohort 2 [*n* = 24]: 24 males, age = 41 ± 12 years; BMI = 26.0 ± 2.1 kg/m^2^), received once-daily sc administrations of either 0.4 (cohort 1) or 0.6 U/kg (cohort 2) Gla-300 for 8 days in one treatment period and Deg-100 for 8 days in the other. A 30-h euglycemic clamp procedure was performed after each 8-day period with blood samples collected at 0, 1, 2, 4, 6, 8, 10, 12, 14, 16, 20, 24, 28, and 30 h after dosing on day 8. A validated radioimmunoassay was used for measurement of total insulin concentration, accounting for both bound and unbound insulins to albumin, with a lower limit of quantification of 12 μU/mL. For more information about the protocol, we refer to Bailey et al.^14^

#### Mathematical model

As reported in the literature,^5–7^ insulin degludec in plasma is present in two rapidly equilibrating forms: an albumin-bound nonactive and a free active form, with a ratio between the two of 0.98–0.99.^6^ Here, we assumed that the two forms are in dynamic equilibrium in plasma, and thus, the ratio between the two may be assumed constant. We calculated an estimate for this ratio from the data reported in Bailey et al.^14^ In particular, for both insulins, we normalized their clinical efficacy, as measured in a euglycemic clamp study by the area under the curve of the 24-h glucose infusion rate (GIR-AUC_0–24h_), to the respective 24-h insulin exposure (Ins-AUC_0–24h_). Then, the efficacy ratio between Deg-100 versus Gla-300 can be determined by comparing the PK-normalized clinical efficacy of Deg-100 to Gla-300 as follows:


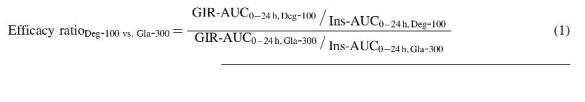


An average efficacy ratio of 0.0285 was found in this population, suggesting a free and active fraction of insulin Deg-100 of 2.85% and an albumin bound fraction of 97.15%. This factor allows us to rescale the plasma concentration of insulin Deg-100 from total to free active and handle it as we did for the other insulin analogues. We acknowledge that this approach for calculating the free and active concentration of Deg-100 may also intrinsically account for any possible difference in the intrinsic activity between the insulins. However, any uncertainity in this scaling factor would only have an effect on the predicted optimal dose for Deg-100 but does not affect the simulated CGM.

The model of sc absorption of free active insulin Deg-100 is three-compartment (Eq. 2):


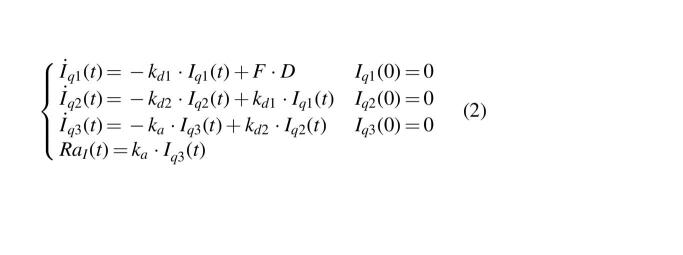


where *D* (mU/kg/min) is the insulin dose administered into the subcutis, *F* (dimensionless) is the bioavailability, *k_d1_* and *k_d2_* (min^−1^) are the rate constants of molecular complex conversion, *k_a_* (min^−1^) is the rate constant of insulin absorption to plasma, and *Ra_I_* is the insulin rate of appearance in plasma.

#### Model identification

As done in Visentin et al.,^19^ the model described in Eq. (2) was coupled with the two-compartment insulin kinetic model of the T1DS^17^ to predict insulin concentration in plasma (Eq. 3):


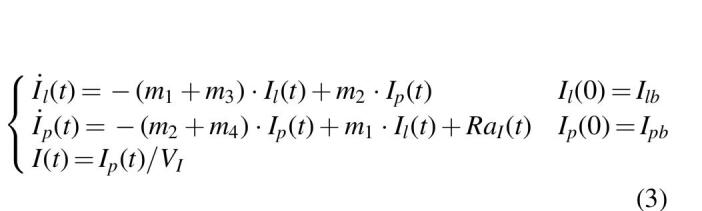


where *I_p_* and *I_l_* (mU/kg) are the mass of free insulin Deg-100 in plasma and liver, respectively, *I* (μU/mL) plasma insulin concentration, suffix *b* denotes basal state, *m_1_*, *m_2_*, *m_3_*, *m_4_* (min^−1^) rate parameters, and *V_I_* (L/kg) the insulin distribution volume.

The model of Eqs. (2–3) is a priori nonidentifiable.^27^ Thus, additional assumptions were needed to make the model uniquely identifiable. In particular, we first assumed *V_I_* fixed to population values (*V_I_* = 0.048 L/kg) and, then, we reparametrized the model so that *m_2_*, *m_3_*, *m_4_* become function of *m_1_*, the plasma insulin clearance (CL, L/min), and *V_I_*, according to what was done in Visentin et al.^19^ and Dalla Man et al.^28^ In addition, due to the experimental setting, during the identification process, the two rate constants *k_d1_* and *k_d2_* were virtually identical in all subjects. Therefore, to improve numerical identifiability, we constrained them to be equal (*k_d_* = *k_d1_* = *k_d2_*). Therefore, five parameters (*F, k_d_, k_a_, m_1_,* CL) were estimated from the data using a maximum a posteriori Bayesian estimator, implemented in MATLAB^®^ R2016b.^29^ A priori information on parameters *m_1_* and CL (*m_1_* = 0.18 min^−1^, CL = 1.11 L/min) was taken from Dalla Man et al.^28^ Measurement error on free insulin Deg-100 data was assumed independent, Gaussian, with zero mean and known variance.^30^

### Incorporation of insulin Deg-100 in the T1DS

The model of Eq. (2) was finally incorporated in the T1DS^17^ by equipping each *in silico* subject with a set of five parameters describing the sc absorption of insulin Deg-100. These were randomly extracted from the joint parameter distribution obtained from model identification in the Deg-100 insulin data reported in Bailey et al.^14^

The validity of the generated PK intersubject variability was assessed by simulating an 8-day repeated administration of both Gla-300 and Deg-100 at 0.4 and 0.6 U/kg, respectively, and by comparing the simulated plasma insulin against their respective clinical counterparts in terms of maximum insulin concentration (*C*_max_) and its timing (*T*_max_).

### *In silico* trial

An *in silico* head-to-head trial was performed to compare glucose control in the 100 virtual subjects receiving Gla-300 versus Deg-100. Like in Visentin et al.,^19^ the average starting basal insulin dose (*b_0_*) and the fasting plasma glucose (FPG) values of the virtual population were matched to baseline values reported in Bergenstal et al.^31^ for Gla-300 (*b_0_* = 0.3 U/kg and FPG = 172.9 mg/dL). For Deg-100, the starting basal insulin dose was properly adjusted to achieve the same baseline FPG levels obtained with Gla-300.

During the trial, virtual subjects were uptitrated to their optimal insulin dose during an 8-week period followed by 4 weeks of stable dosing. For both Gla-300 and Deg-100 treatment arms, both morning (immediately before breakfast until lunch) and evening (immediately before the evening meal until bedtime) insulin dose administrations were simulated. In addition, two different titration rules were tested for both injection schedules and insulin formulations, as detailed below.

As done in Visentin et al.,^19^ during each 8-week period, virtual subjects received three meals per day, each accompanied by its optimal prandial insulin bolus. Meal attributes, that is, timing and amount, were generated mimicking the habits occurring in real life, as described in Vettoretti et al.^32^ and Visentin et al.^33^ Optimal prandial insulin bolus (B [U]) was determined from subject's preprandial glycemia (G [mg/dL]), carbohydrate content of the meal (CHO [g]), insulin on board (IOB [U]), and subject-specific insulin therapy parameters, such as CR [g/U], CF [mg/dL/U], and G_target_ [mg/dL]. In particular, G_target_ was fixed to 160 mg/dL as in Bergenstal et al.,^31^ IOB was modeled as in Hu and Li,^34^ and a percentage error on carbohydrate counting was added as described in Vettoretti et al.^32^

Basal insulin dose was titrated every 7 days to achieve the target prebreakfast blood glucose (BG). For both insulin formulations and injection schedules, two different basal insulin titration rules were implemented, with titration rules A and B derived from Home et al.^11^ and Heller et al.,^35^ respectively. In particular, virtual patients were titrated to their optimal individual dose until they reached the glucose target, which is 80–130 mg/dL for titration rule A and 70–89 mg/dL for titration rule B (more details about titration rules are reported in Supplementary Table S1). In addition, a hypoglycemia-related stop condition has been introduced for the uptitration of each individual patient. Based on the results reported in Bergenstal et al.,^31^ we chose to tolerate 7.5% of the time spent below 70 mg/dL at maximum.

Finally, an interoccasion variability in Gla-300 and Deg-100 bioavailability was generated by randomly modulating the subject-specific nominal value with a Gaussian noise with zero mean and coefficient of variation equal to 17% for Gla-300^36^ and 8.5% for Deg-100.^13^

### Outcome metrics and statistical analysis

Efficacy of glucose control was assessed by calculating the standard outcome metrics proposed in Danne et al.^37^ from the last 2 weeks of CGM data. In particular, the primary outcome was the mean percentage of time within 70–140 mg/dL (*T*_t,70–140_), while secondary outcomes include mean and standard deviation (SD) of glucose excursion, percentage of time within glucose ranges <54 (*T*_b,54_), 70–180 (*T*_t,70–180_), >180 (*T*_a,180_), >250 (*T*_a,250_) mg/dL, low blood glucose index (LBGI), and high blood glucose index (HBGI).^38^ These metrics were calculated on a daily basis from individual CGM profile, for each treatment effect and insulin formulation, and averaged among the last 2 weeks of experiment. In addition, the coefficient of variation of glucose excursion was calculated on a 2-week basis (CV_2-weeks_).

Model parameter estimates were reported as median (25th–75th) percentiles, with precision expressed as median percent coefficient of variation (CV). As in Danne et al.,^37^ outcome metrics were reported as mean ± SD. Comparison was performed based on parameter distribution: *t*-test (paired for the “crossover” and unpaired for the “parallel” design, see below) for normally distributed values, and nonparametric test (Wilcoxon for the “crossover” and Mann–Whitney *U* for the “parallel” design, see below) otherwise. The normality of distributions was assessed by the Lilliefors test. Significance level was set at *P* = 0.05 for all the statistical tests.

As previously described, the simulation framework allows to perform the same experimental scenario to the whole virtual population, hence simulating a “crossover” design. However, since in a clinical trial, a “parallel” design will likely be preferred for practical reason, here both design settings have been simulated.

In the “crossover” design, we performed the paired comparison between the two insulins, for both titration rules and injection schedules. In the “parallel” design, the virtual population was randomly split in two equal subgroups (*n* = 50 subjects each), that is, 50 subjects for the Gla-300 group and the remaining 50 subjects for the Deg-100 group, and the unpaired comparison, for each titration rule and injection schedule, was performed. The random splitting of the virtual population in the two treatment groups and the corresponding statistical analysis were repeated 100-fold for each statistical outcome. This allowed assessing the robustness of the statistical outcomes achievable by the “parallel” versus “crossover” design, by quantifying how many times a statistically significant difference is detected among the 100 random extractions.

## Results

### Assessment of Deg-100 PK model

**FIG. 1.**
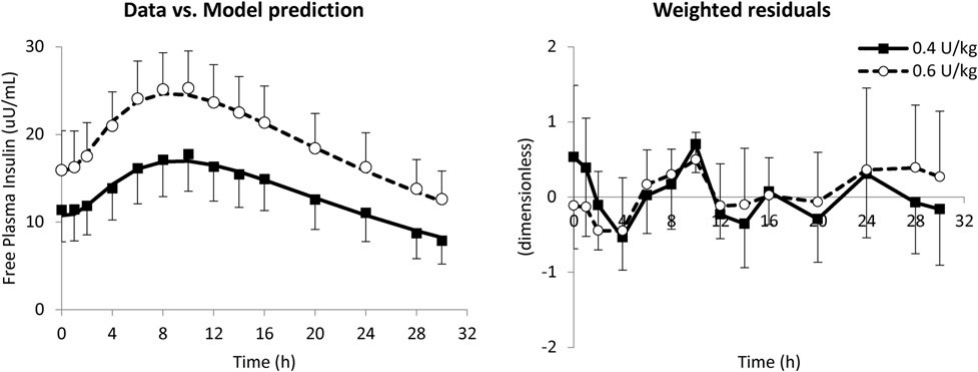
Mean ± SD of data versus model prediction (left) and weighted residuals (right panel) at 0.4 (white circles and dashed line) and 0.6 U/kg (black squares and continuous line). SD, standard deviation.

On average, the model well predicted plasma free insulin Deg-100 data (Fig. 1, left panel) for both 0.4 and 0.6 U/kg doses (black squares and white circles, respectively), as confirmed by the weighted residuals, which were sufficiently random and laid within the ±1 interval (Fig. 1, right panel). Model parameters were estimated with good precision, and no statistically significant differences were found between administration doses: *F* = 69 [58–85]% (CV = 34%), *k_d_* = 0.0056 [0.0041–0.0072] min^−1^ (22%), *k_a_* = 0.0007 [0.0006–0.0009] min^−1^ (20%), *m_1_* = 0.176 [0.176–0.177] min^−1^ (50%), and CL = 1.10 [1.09–1.11] L/min (34%).

**FIG. 2.**
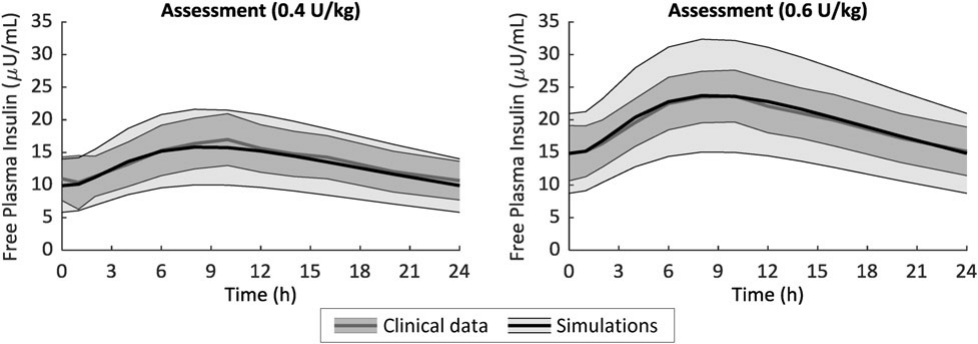
Mean ± SD of free-active (see text for more details) simulated (black line) versus clinical (gray line) plasma insulin obtained after 8-day administration of Deg-100 at 0.4 U/kg (left) and 0.6 U/kg (right panel). Deg-100, degludec 100 U/mL.

One hundred *in silico* Deg-100 PK parameter vectors have been generated from the multivariate parameter distribution and incorporated into the simulator, enabling to simulate the PK of insulin Deg-100. After 8 days of insulin degludec administration at 0.4 and 0.6 U/kg, the simulated plasma insulin time courses well reproduced the data, as shown in Figure 2. Maximum insulin concentration (*C*_max_) and its timing (*T*_max_) were, respectively, 16.0 ± 5.8 μU/mL and 538 ± 97 min versus 17.0 ± 4.0 μU/mL and 565 ± 56 min for simulated versus clinical data at 0.4 U/kg, while 24.0 ± 8.7 μU/mL and 538 ± 97 min versus 24.1 ± 4.1 μU/mL and 512 ± 159 min for simulated versus clinical data at 0.6 U/kg. No statistically significant differences were found between measured and simulated profiles.

### In silico trial

#### “Crossover” design

**FIG. 3.**
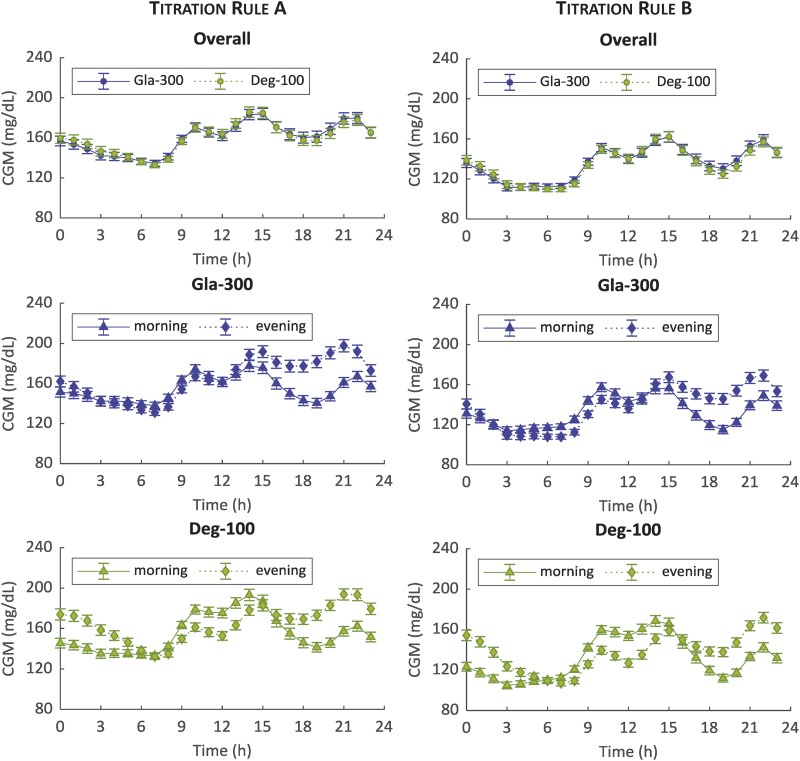
Mean ± standard error of 100 simulated CGM glucose profiles over 24 h during the last 2 weeks for titration rules A (left) and B (right) and morning (triangle) versus evening (diamond) injections: Gla-300 (blue) versus Deg-100 (green) overall (top), Gla-300 by injection schedule (middle) and Deg-100 by injection schedule (bottom panels). CGM, continuous glucose monitoring; Gla-300, glargine 300 U/mL. Color images are available online.

The simulated average 24-h glucose profiles of 100 virtual patients under Gla-300 versus Deg-100 for titration rules A and B are shown in Figure 3. Overall, i.e. averaging results of morning and evening dosing, the glucose control achieved using the two insulins is almost superimposable on average (Fig. 3, top panels), with lower glucose values obtained with titration rule B (135.7 ± 21.7 mg/dL vs. 134.7 ± 21.3 mg/dL, Gla-300 vs. Deg-100, respectively; *P* = 0.77) than titration rule A (160.0 ± 23.5 mg/dL vs. 160.1 ± 21.9 mg/dL, Gla-300 vs. Deg-100, respectively; *P* = 0.21) likely due to the lower prebreakfast BG target aimed by titration rule B (Supplementary Table S1).

For both insulins and titration rules, a better glucose control is achieved during afternoon and dinner with the morning versus evening administration. However, Gla-300 achieves a better glucose control during night with evening versus morning administration for both titration rules (Fig. 3, middle panels), while the opposite occurs for Deg-100 (Fig. 3, bottom panels) most likely due to the Deg-100 PK profile with a *T*_max_ around 11 h.

The comparison, in terms of outcome metrics, between Gla-300 and Deg-100 is reported in Table 1. Glucose control, expressed as mean percentage of time within the range of 70–140 mg/dL (primary outcome), was similar between the two insulins for both titration rule A (39.5% ± 16.4% vs. 38.9% ± 15.9%, Gla-300 and Deg-100 respectively; *P* = 0.26) and titration rule B (56.8% ± 16.8% vs. 57.4% ± 17.4%, Gla-300 and Deg-100, respectively; *P* = 0.95). Although both insulins show no statistically significant difference in the primary outcome, differences between Gla-300 versus Deg-100 are found in some secondary outcomes, depending upon the injection schedule.

**Table 1.** “Crossover” Design: Outcome Metrics and Statistical Analysis

Metric	Dosing regimen	Titration rule A			Titration rule B		
		Gla-300	Deg-100	P	Gla-300	Deg-100	P
Mean (mg/dL)	Morning	154.8 ± 22.7	155.4 ± 21.4	0.37	133.1 ± 21.6	131.6 ± 20.5	0.47
	Evening	165.2 ± 23.2	164.8 ± 21.6	0.39	138.2 ± 21.6	137.7 ± 21.8	0.78
SD (mg/dL)	Morning	38.0 ± 11.2	40.8 ± 12.0	<0.001	35.4 ± 9.8	38.0 ± 11.1	<0.001
	Evening	43.2 ± 14.6	41.2 ± 11.8	0.10	39.2 ± 12.7	38.0 ± 10.2	0.78
CV_2-weeks_ (%)	Morning	27.9 ± 5.0	28.8 ± 5.8	0.004	30.0 ± 5.2	31.4 ± 6.1	<0.001
	Evening	29.1 ± 5.8	27.8 ± 5.0	<0.001	31.5 ± 6.1	30.4 ± 5.3	0.023
*T*_b,54_ (%)	Morning	0.2 ± 0.5	0.2 ± 0.5	0.60	0.7 ± 0.9	0.8 ± 0.9	0.45
	Evening	0.1 ± 0.3	0.1 ± 0.2	0.17	0.6 ± 0.7	0.5 ± 0.6	0.21
*T*_b,70_ (%)	Morning	1.3 ± 1.9	1.4 ± 2.0	0.61	4.2 ± 3.4	4.7 ± 3.6	0.14
	Evening	0.7 ± 1.1	0.6 ± 0.9	0.06	3.6 ± 3.2	3.3 ± 2.6	0.13
*T*_t,70–140_ (%)	Morning	42.5 ± 17.3	42.7 ± 15.9	0.93	58.5 ± 17.5	59.2 ± 16.7	1
	Evening	36.4 ± 14.9	35.1 ± 15.0	0.14	55.1 ± 16.0	55.6 ± 17.9	0.75
*T*_t,70–180_ (%)	Morning	73.4 ± 16.0	72.6 ± 15.2	0.19	81.2 ± 11.9	80.8 ± 11.0	0.29
	Evening	67.1 ± 16.0	67.4 ± 16.3	0.92	78.9 ± 12.7	79.5 ± 13.3	0.43
*T*_a, 180_ (%)	Morning	25.3 ± 16.4	26.0 ± 15.5	0.20	14.6 ± 13.1	14.6 ± 11.9	0.64
	Evening	32.2 ± 16.2	32.0 ± 16.5	0.75	17.5 ± 13.6	17.2 ± 14.4	1
*T*_a, 250_ (%)	Morning	4.7 ± 6.6	5.1 ± 6.3	0.14	2.2 ± 3.7	2.2 ± 3.6	0.21
	Evening	7.7 ± 9.2	6.6 ± 7.5	0.65	3.3 ± 5.4	2.6 ± 3.9	0.70
LBGI (%)	Morning	0.41 ± 0.44	0.43 ± 0.48	0.76	1.19 ± 0.82	1.33 ± 0.85	0.032
	Evening	0.27 ± 0.27	0.23 ± 0.22	0.015	1.05 ± 0.73	0.99 ± 0.65	0.15
HBGI (%)	Morning	5.66 ± 3.52	5.81 ± 3.33	0.15	3.33 ± 2.60	3.30 ± 2.45	0.74
	Evening	7.29 ± 4.04	7.04 ± 3.61	0.83	4.02 ± 3.01	3.85 ± 2.79	0.98

Mean ± SD of outcome metrics obtained with Gla-300 and Deg-100. All metrics were calculated on a daily basis of the individual continuous glucose monitoring profile, except for CV_2-weeks_, which was calculated on a 2-week basis. Comparison was performed based on parameter distribution: paired *t*-test for normally distributed values, and Wilcoxon signed-rank test otherwise. Significance level was set at *P* = 0.05 (shaded gray area highlights statistically significant values).

CV, coefficient of variation; Deg-100, degludec 100 U/mL; Gla-300, glargine 300 U/mL; HBGI, high blood glucose index; LBGI, low blood glucose index; SD, standard deviation.

In particular, when administered in the morning, virtual patients with Gla-300 showed a lower glucose variability, both within-day (SD^Gla-300^–SD^Deg-100^: −2.8 mg/dL, *P* < 0.001 and −2.6 mg/dL, *P* < 0.001 for titration rules A and B, respectively) and day-to-day (CV_2-weeks_^Gla-300^–CV_2-weeks_^Deg-100^: −0.9%, *P* = 0.009 and −1.4%, *P* < 0.001 for titration rules A and B, respectively), together with a lower LBGI (LBGI^Gla-300^–LBGI^Deg-100^: −0.02, *P* = 0.76 and −0.14, *P* = 0.032 for titration rules A and B, respectively). Conversely, when administered in the evening, virtual patients with Deg-100 showed a lower day-to-day glucose variability (CV_2-weeks_^Deg-100^–CV_2-weeks_^Gla-300^: −1.4%, *P* < 0.001 and −1.1%, *P* = 0.023 for titration rules A and B, respectively) and LBGI (LBGI^Deg-100^–LBGI^Gla-300^: −0.04, *P* = 0.015 and −0.06, *P* = 0.15 for titration rules A and B, respectively).

In addition, the number of virtual patients with CV_2-weeks_ lower than 36%^37^ was slightly lower for Gla-300 versus Deg-100, when injected in the evening (titration rule A: 88% vs. 95%, for Gla-300 vs. Deg-100, respectively; titration rule B: 79% vs. 82%, for Gla-300 vs. Deg-100, respectively), while it was higher for Gla-300 versus Deg-100 with morning administration (titration rule A: 96% vs. 89%, for Gla-300 vs. Deg-100, respectively; titration rule B: 86% vs. 72%, for Gla-300 vs. Deg-100, respectively).

Finally, for both titration rules, average stable doses at the end of each treatment period were significantly lower (*P* < 0.001) for Gla-300 (0.43 ± 0.13 U/kg and 0.56 ± 0.14 U/kg for titration rules A and B, respectively) versus Deg-100 (0.38 ± 0.13 U/kg and 0.51 ± 0.13 U/kg for titration rules A and B, respectively). In particular, average doses resulted slightly higher when administered in the morning than evening both for Gla-300 (Gla-300^morning^–Gla-300^evening^: 0.04 ± 0.06 U/kg, *P* < 0.001, and 0.02 ± 0.06 U/kg, *P* = 0.01, for titration rules A and B, respectively) and Deg-100 (Deg-100^morning^–Deg-100^evening^: 0.02 ± 0.06 U/kg, *P* = 0.009, and 0.01 ± 0.07 U/kg, *P* = 0.45, for titration rules A and B, respectively).

#### “Parallel” design

In addition to simulating the “crossover” design, i.e. comparing each treatment in the same virtual patient described in the paragraph above, here we also analyzed the simulation results assuming a “parallel” design. In particular, we randomly split the virtual population in the two treatment groups of 50 patients each and we performed the corresponding statistical analysis for each statistical outcome. Moreover, we performed this random splitting of the virtual population 100-fold and captured how many times a specific outcome metric showed a statistically significant difference between treatment groups (Supplementary Table S2).

In particular, a statistically significant difference in the primary outcome, for both titration rules and injection schedules, was found in <5% of the times. However, in some of the secondary outcomes, a slightly higher occurrence of a statistically significant difference has been observed. In particular, when administered in the morning, Gla-300 showed a lower within-day variability than Deg-100 in 17% and 15% of the cases (titration rules A and B, respectively), together with a lower between-day variability in 11% and 20% of the cases (titration rules A and B, respectively). In contrast, when administered in the evening, Deg-100 showed a lower between-day variability than Deg-100 in 21% and 11% of the cases (titration rules A and B, respectively). In summary, assuming a “parallel” design, in at least 79% of the virtual trials, no statistically significant difference between the two insulins has been found for any of the primary or secondary outcomes.

## Discussion

Long-acting insulin analogues are currently used in MDI therapy for T1D to cover basal insulin needs. Research in the field is very active, with pharma companies working on the development of new basal insulin analogues aiming to reduce both day-to-day and within-day variabilities in BG levels. However, before a new basal insulin can be brought to the patients, its efficacy and safety must be proven in comparison with the existing treatment options in timely and costly clinical trials.

With the development of simulation platforms able to reproduce glucose variability characterizing the T1D population, such as the UVA/Padova T1DS,^17^ clinical head-to-head comparisons of different insulin treatments can be performed to evaluate efficacy and safety in a cost-effective way. In addition, virtual trials can be run multiple times pulling different virtual patients into the trial each time and assessing the probability for showing a statistical difference in any of the clinical endpoints. In addition, as subtle treatment differences often remain “invisible” in clinical trials due to the high patient-to-patient variability (or due to the small patient number in each treatment arm), computer simulations also offer the opportunity to explore different treatment effects and clinical trial designs in the same virtual patient and potentially make subtle differences visible.

In particular, here we performed a virtual trial to compare the glucose control achieved by insulin Gla-300 versus Deg-100 in patients with T1D. We aimed at, based on the findings of a clamp study comparing both insulins,^14^ predicting their clinical efficacy under a more physiological everyday life settings.

Toward this end, a model describing the sc absorption of insulin Deg-100 was developed and incorporated into the UVA/Padova T1DS, in addition to the one already available for insulin Gla-300.^18,19^ The model well fitted free insulin Deg-100 data (Fig. 1) and parameters were estimated with precision. The model was then incorporated into the simulator and individual parameterizations have been randomly generated and assigned to the virtual subjects (Fig. 2). It is worth noting that, in the parameter generation, we took into account the existence of the correlation among model parameters derived from a study where subjects received both Gla-300 and Deg-100.^19^

A head-to-head virtual trial was conducted to compare glucose control in patients with T1D under MDI therapy treated with Gla-300 or Deg-100. In particular, for each insulin, the virtual patient underwent the same experimental protocol, that is, three meals per day with optimal meal insulin bolus, with basal insulins uptitrated to their individual dose for 8 weeks followed by 4 weeks of stable dosing, by either morning or evening injection and two different titration rules.

The simulations show the overall similarity of the 24-h glucose profiles of virtual patients under treatment with Gla-300 or Deg-100: almost superimposable average glucose control for two insulins (Fig. 3, top panels), with no statistically significant difference in the primary outcome, regardless of injection schedules (morning or evening) and titration rules to guide patients to their individual insulin dose. In the “crossover” design, some statistically significant differences in the secondary outcomes are revealed: Gla-300 showed—regardless of the applied titration rule—a lower within- and between-day variability and a lower LBGI when administered in the morning, while Deg-100 showed a lower between-day variability and LBGI when administered in the evening. Average insulin doses in the stable period, that is, after 8 weeks of uptitration, resulted to be lower for Deg-100 than Gla-300, in accordance with the results reported in Bailey et al.^14^

These results were obtained in an *in silico* “crossover” trial that represents the best configuration to do a robust comparison since each patient underwent the same identical experimental protocol except for the insulin treatment. To assess if these differences would be detected also in a more traditionally chosen “parallel design,” we randomly (100-fold) split the virtual population in two equal subgroups and compared the observed differences in the primary and secondary endpoints between groups. As expected, given the reduced statistical power of the “parallel” versus “crossover” design, these differences only became “visible” in the “parallel” design in less than or equal to 21% of the cases, in correspondence with the “crossover” design pointing to statistical significance.

Performing a hypothesis testing requires the definition of the null hypothesis. This is sometimes implicitly set to be “Treatment A is equal to Treatment B,” thus finding a *P* > 0.05 does not necessarily mean that Treatment A is not inferior to Treatment B.^39^ Thus, we performed a noninferiority test on the primary outcome and found that Gla-300 is not worse than Deg-100. In particular, we assumed no difference in the outcome between the two insulins, with a noninferiority margin (Δ) equal to 50% of the SD of the primary outcome for Deg-100 (Δ = 0.5·SD = 8%) and a significance level *α* = 2.5%.

Although this work reveals the great potential of computer simulations to inform or even reduce the number of clinical trials, we would also like to highlight some differences between virtual versus real experiments and the limitations of the first ones. Obviously, the virtual patients are under much more controlled and standardized conditions than the true patients enrolled in a clinical trial. The virtual patient strictly adheres to the titration rule to reach the individual glucose target and to the meal schedule, while the patient enrolled in a clinical trial may not. In addition, patient habits, such as physical activity or “nocturnal snacks” were not included in this study. Thus, the results presented in this work are obtained in ideal conditions since, by definition, the virtual trial allows keeping under control any confounding effect and thus making also small differences visible, which may disappear in a less standardized setting of a clinical trial.

Another limitation is the value for Deg-100 albumin-bound-to-free ratio used in this work, which has been calculated by comparing the PK-normalized clinical efficacy of Deg-100 versus Gla-300 using the data from the euglycemic clamp study reported in Bailey et al.^14^ This approach for calculating the free and active concentration of Deg-100 may intrinsically account for the albumin-bound-to-free ratio of insulin Deg-100 and/or possible different intrinsic activity in Deg-100 versus Gla-300. However, if a different value was assumed for this efficacy factor, the results would be unchanged apart from the optimal dose achieved in each virtual patient. Future work will exploit the assessment of the insulin dosing between the two insulins exploiting the findings of the ongoing clinical trials comparing the two insulins to better reproduce the optimal insulin doses of the two insulin analogues.

In conclusion, as the clinical trial may be seen as a model for the real world, we believe that a virtual trial can be seen as a model for a clinical trial and thus is an important means for its experimental design. As a matter of fact, the results of this work have been used to support and inform the design of a clinical trial, for example, time of dosing, glucose target, and titration rule, comparing insulin Gla-300 versus Deg-100 in patients with T1D using a CGM-based metric as a clinical endpoint (NCT04075513).

Models are indeed simplified versions of the real world but, if properly developed and used, they can be of help in reducing costs and time both in the design and in product development.^40^

## Conclusions

In this work, an *in silico* trial was performed aiming to inform and derisk the design of a head-to-head clinical trial comparing glucose control in patients with T1D receiving Gla-300 or Deg-100. Different titration rules as well as dosing schedules have been explored in crossover and parallel trial designs. Toward this end, a model of sc absorption of Deg-100 was developed and incorporated into the UVA/Padova T1DS, which already included a PK model of Gla-300. Overall, the results of the *in silico* trial suggest a similar efficacy of Gla-300 and Deg-100 in the main endpoint, the time with glucose in the range of 70–140 mg/dL. The applied methodology could become a new paradigm for the design of clinical trial testing of novel insulins as well as for optimizing titration rules and dosing regimens for those already on the market.

## Supplementary Material

Supplemental data
