# Supplementary material for: In Silico Head-to-Head Comparison of Insulin Glargine 300 U/mL and Insulin Degludec 100 U/mL in Type 1 Diabetes
Source: Diabetes Technol Ther. 2020 Jul 27;22(8):553–61. doi: 10.1089/dia.2020.0027 (PMC7407002; doi:10.1089/dia.2020.0027)
Supplement: Supplemental data [file Supp_TableS1-S2.pdf]

Supplementary Data

Supplementary Table S1. Titration Rules Implemented in the In Silico Trial for Both Insulin Formulations and Injection Schedules

Titration rule A		*Titration rule B*	
Dose increment [U]	*Median last 3 days prebreakfast BG [mg/dL]*	*Dose increment [U]*	*Mean last 3 days prebreakfast BG [mg/dL]*
−1.5	<80	−4	<56
		−2	56–69
0	80–130	0	70–89
+1.5–4.0[a]	>130–249	+2	90–179
		+4	180–269
+4.5	≥250	+6	≥270

Shaded gray area corresponds to prebreakfast BG target.
[a]0.5 U step every 20 mg/dL.
BG, blood glucose.

Supplementary Table S2. "Parallel" Design: Occurrence of Statistically Significant Difference ($P < 0.05$)

		Titration rule A		*Titration rule B*	
Metric	*Dosing regimen*	*Gla-300 > Deg-100 (%)*	*Deg-100 > Gla-300 (%)*	*Gla-300 > Deg-100 (%)*	*Deg-100 > Gla-300 (%)*
Mean (mg/dL)	Morning	2	3	1	0
	Evening	1	3	2	1
SD (mg/dL)	Morning	0	17	0	15
	Evening	4	0	1	1
$CV_{2\text{-weeks}}$ (%)	Morning	0	11	0	20
	Evening	21	0	11	0
$T_{b,54}$ (%)	Morning	0	2	0	3
	Evening	3	1	3	0
$T_{b,70}$ (%)	Morning	3	1	0	8
	Evening	5	1	0	0
$T_{t,70–140}$ (%)	Morning	1	1	0	2
	Evening	3	0	2	3
$T_{t,70–180}$ (%)	Morning	2	1	3	0
	Evening	1	2	1	3
$T_{a,\ 180}$ (%)	Morning	1	2	0	1
	Evening	2	1	2	1
$T_{a,\ 250}$ (%)	Morning	0	5	0	1
	Evening	4	0	4	0
LBGI (%)	Morning	1	1	0	9
	Evening	4	1	2	0
HBGI (%)	Morning	1	2	1	1
	Evening	4	1	3	1

Percentage of occurrence of statistically significant difference ($P < 0.05$) in outcome metrics between Gla-300 versus Deg-100 among the 100 random extractions ($n = 50$ per group). All metrics were calculated on a daily basis of the individual continuous glucose monitoring profile, except for $CV_{2\text{-weeks}}$, which was calculated on a 2-week basis. Comparison was performed based on parameter distribution: unpaired *t*-test for normally distributed values, and Mann–Whitney *U* test otherwise.

CV, coefficient of variation; Deg-100, degludec 100 U/mL; Gla-300, glargine 300 U/mL; HBGI, high blood glucose index; LBGI, low blood glucose index; SD, standard deviation.
